# *rGREAT*: an R/bioconductor package for functional enrichment on genomic regions

**DOI:** 10.1093/bioinformatics/btac745

**Published:** 2022-11-17

**Authors:** Zuguang Gu, Daniel Hübschmann

**Affiliations:** Molecular Precision Oncology Program, National Center for Tumor Diseases (NCT), Heidelberg 69120, Germany; Molecular Precision Oncology Program, National Center for Tumor Diseases (NCT), Heidelberg 69120, Germany; Heidelberg Institute of Stem Cell Technology and Experimental Medicine (HI-STEM), Heidelberg 69120, Germany; German Cancer Consortium (DKTK), Heidelberg 69120, Germany; Department of Pediatric Immunology, Hematology and Oncology, University Hospital Heidelberg, Heidelberg, 69120, Germany

## Abstract

**Summary:**

*GREAT* (Genomic Regions Enrichment of Annotations Tool) is a widely used tool for functional enrichment on genomic regions. However, as an online tool, it has limitations of outdated annotation data, small numbers of supported organisms and gene set collections, and not being extensible for users. Here, we developed a new R/Bioconductorpackage named *rGREAT* which implements the *GREAT* algorithm locally. *rGREAT* by default supports more than 600 organisms and a large number of gene set collections, as well as self-provided gene sets and organisms from users. Additionally, it implements a general method for dealing with background regions.

**Availability and implementation:**

The package *rGREAT* is freely available from the Bioconductor project: https://bioconductor.org/packages/rGREAT/. The development version is available at https://github.com/jokergoo/rGREAT. Gene Ontology gene sets for more than 600 organisms retrieved from Ensembl BioMart are presented in an R package *BioMartGOGeneSets* which is available at https://github.com/jokergoo/BioMartGOGeneSets.

**Supplementary information:**

Supplementary data are available at *Bioinformatics* online.

## 1 Introduction

Genomics and epigenomics studies often generate many lists of genomic regions of interest, e.g. single-nucleotide variants (SNVs) from whole genome sequencing or exome sequencing data, peak regions of a certain chromatin modification from ChIP-sequencing data, or differentially methylated regions (DMRs) from whole genome bisulfite sequencing data. The next step of analysis is naturally to associate biological functions with these genomic regions. A widely used approach is to first annotate genomic regions to the nearest genes, then to apply over-representation analysis (ORA) on the genes against a collection of gene sets where each gene set corresponds to a specific biological function. ORA is a commonly used gene set enrichment analysis approach for analyzing whether a list of genes, e.g. differentially expressed genes, are enriched in a gene set (Khatri and Drăghici, 2005). However, in the context of genomic regions, applying ORA directly to genes might not be an appropriate approach. In ORA where the enrichment analysis is normally performed by Fisher’s exact test or based on hypergeometric distribution, the null assumption is that genes are independent and they have the same probability to be picked; but when dealing with genomic regions, the null assumption becomes ‘genomic regions are uniformly distributed on the genome’. Due to the fact that genes are not equally distributed on the genome and genes have different lengths, conversion from genomic regions to genes leads to genes not being picked with equal probability. A gene has a low probability to be picked when regions are all far from it. As a comparison, a gene is more likely to be picked when there is a cluster of regions close to it. A gene is also more likely to be picked when the gene has a larger length. These scenarios violate the null assumption of ORA and would produce false positives and improper enrichment results on genomic regions.

**Fig. 1.**
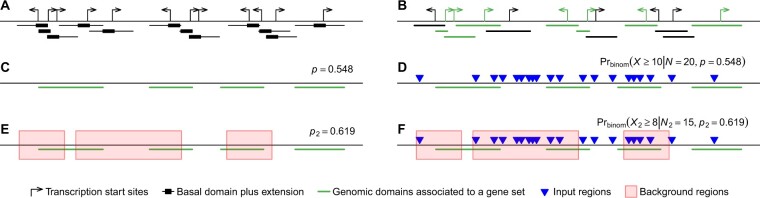
The binomial model of the *GREAT* analysis. (**A**) Basal domain and extensions around transcription start sites of genes. (**B**) A region set which is associated with genes in a specific gene set (green segments). The basal domain and its extensions are reduced as a single segment in the figure. (**C**) Overlapping regions in the region set are merged. The fraction of the genome that is covered by the region set is defined as *p*. (**D**) For a list of *N* input regions, the number of input regions that fall into the region set follows a binomial distribution. (**E**) When background regions are provided, the fraction of the background regions that is covered by the region set (within the red rectangles) is denoted as *p*_2_. (**F**) For a list of *N* input regions, only *N*_2_ input regions that fall into the background are considered. The number of input regions that fall in both region set and background also follows a binomial distribution. The figures are adapted from the original *GREAT* paper (A color version of this figure appears in the online version of this article)

The tool *GREAT* (Genomic Regions Enrichment of Annotations Tool) (McLean *et al.*, 2010) was developed in 2010. The initial aim of *GREAT* was to associate biological functions to cis-regulatory elements, e.g. transcriptional factor binding sites (TFBS), but its algorithm allows it to be extended to any type of genomic regions. Instead of the gene-centric enrichment of ORA, *GREAT* converts the problem to region centric. For a gene in a given gene set, a basal domain extending its transcription start site (TSS), e.g. to upstream 5 kb and downstream 1 kb, is firstly established which captures the TSS-related short-range associations; next the basal domain is extended in both directions to maximal 1 mb or until it reaches the neighbor gene’s basal domain, which captures the distal associations (Fig. 1A). In this way, for genes in the gene set, a list of extended TSSs is constructed and they are associated with the biological function of this gene set (Fig. 1B). Simply speaking, *GREAT* directly constructs such ‘region sets’ (or genomic domains) that associate with individual biological functions. The enrichment test is applied as follows. For a specific biological term represented as a gene set, denote the fraction of its associated functional domains in the genome (Fig. 1C) as *p*, the total number of input regions as *N*, the observed number of input regions that fall in the associated domains as *n* and the corresponding random variable as *X*, then *X* follows a binomial distribution: *X* ∼ B(*p*, *N*) and the *P*-value of the enrichment is calculated as Pr(*X *≥* n*) (Fig. 1D).

*GREAT* has been widely applied in a large number of studies, nevertheless, there are limitations with respect to applicability for the users. As an online tool, all annotation resources are only controlled by *GREAT* developers; they are not extensible by users. The current version (4.0.4) of *GREAT* only supports human and mouse, and it only supports seven gene set collections which have not yet been updated to the most recent ones. In this work, we present a R/Bioconductor package named *rGREAT*. It applies *GREAT* analysis in two ways. First, it serves as a client to directly interact with the *GREAT* web service in the R environment. It automatically submits the input regions to *GREAT* and retrieves results from there. Second, it implements the *GREAT* algorithm locally, and it is seamlessly integrated with the Bioconductor annotation ecosystem. On one hand, with local *GREAT*, it is possible to perform enrichment analysis on any organism and with any type of gene set collection; and on the other hand, Bioconductor annotation packages are well maintained and updated, which ensures that a local *GREAT* analysis always uses the most up-to-date annotation data. Local *GREAT* by default supports many gene set collections and more than 600 organisms, and, more importantly, local *GREAT* allows self-provided gene sets and organisms from users.

## 2 Methods and results

### 2.1 Online GREAT

*rGREAT* supports interaction with the *GREAT* web service programmatically. The function submitGreatJob() automatically submits the input regions to the *GREAT* web service, the function getEnrichmentTables() retrieves results from *GREAT*, and the function plotRegionGeneAssociations() generates plots of associations between input regions and genes that are the same as those from the *GREAT* web service. submitGreatJob() supports all historical versions of *GREAT*.

### 2.2 Local GREAT

The function great() implements the *GREAT* algorithm and applies *GREAT* analysis locally with a specific gene set collection on a specific organism. great() has integrated Gene Ontology (GO) gene sets for all supported organisms and MSigDB gene sets (Liberzon *et al.*, 2011) for humans. great() supports more than 600 organisms; it furthermore allows users to integrate self-provided gene sets and organisms through a simple application programming interface. The enrichment results can be viewed via a Shiny web application with the function shinyReport().

### 2.3 Working with background regions

*GREAT* applies a different enrichment test when background regions are provided. If denoting background regions as a list of *n* intervals (*x_i_*, *y_i_*) with index set *A* = {1, …, *n*}, the input regions can only be a list of intervals (*x_j_*, *y_j_*) of which the corresponding index set *B* is a subset of *A*. In this setting, for a biological term, *GREAT* counts the number of background regions denoted as *N*_bg_, the number of input regions (or foreground regions) denoted as *N*_fg_, the number of background regions that fall in the associated functional domains denoted as *n*_bg_, the number of input regions that fall in the associated domains as *n*_fg_ and the corresponding random variable as *X*_fg_, then *X*_fg_ follows a hypergeometric distribution: *X*_fg_ ∼ Hyper(*N*_bg_, *N*_fg_, *n*_bg_). The *P*-value is calculated as Pr(*X*_fg_ ≥ *n*_fg_). A similar strategy is also implemented in the *LOLA* package (Sheffield and Bock, 2016).

This approach is useful in various scenarios. For example, for a TF whose binding sites are measured by ChIP sequencing, the union of its peaks from all tissues can be taken as the background set, and peaks from one specific tissue are taken as the input region set, then we can test which biological functions are enriched for tissue-specific TFBS peaks against the background set. However, such background sets sometimes are not easy to obtain because the background regions must be a super set of input regions and they should share the same biological measurements. In addition, researchers may also consider different aspects of this ‘background’. For example, they may want to exclude assembly gap regions or unsequenced regions from the analysis. The null assumption of the *GREAT* binomial model is that input regions are uniformly distributed across the genome. Since the unsequenced regions are never measured, it is highly advisable to exclude them from an analysis (Domanska *et al.*, 2018). In Supplementary File S1, we demonstrated that indeed excluding gap regions decreases the number of enriched terms. Other example scenarios of such types of backgrounds are: when analyzing DMRs, the background can be set as regions showing similar CpG densities as the input DMRs; or to only consider autosomes when gender leads to a huge batch effect in the analysis. A proper background should be selected based on the attributes of the input regions and the specific biological questions to answer. A large and improper background normally underestimates the fractions of biological term-associated domains in the genome and generates lower *P*-values, thus possibly more false positives (Supplementary File S1).

For setting a proper background, great() supports two arguments: background and exclude. background accepts pre-selected background regions and exclude accepts regions that will be excluded from the genome. If any of the two arguments is set, the input regions and the extended TSSs are intersected with the background, and the *GREAT* binomial model is only applied to the reduced regions. When background regions are set, for a biological term, let *N*_2_ denote the total number of input regions that overlap the background, let *p*_2_ be the fraction of the associated functional domains only in the background (Fig. 1E), let *n*_2_ denote the observed number of input regions that fall in the associated domains restricted by the background and let the corresponding random variable be denoted as *X*_2_, then *X*_2_ follows the binomial distribution *X*_2_ ∼ B(*p*_2_, *N*_2_) and *P*-values are calculated as Pr(*X*_2_ ≥ *n*_2_) (Fig. 1F). In fact, the hypergeometric method in *GREAT* can be approximated to the binomial method here. Following the denotations used previously, there are *N*_2_ ≡ *N*_fg_ and *n*_2_ ≡ *n*_fg_. If regions do not overlap, we can conclude

$$p_{2}=\sum_{i\;\in \;B}w_{i}/\sum_{i\;\in \;A}w_{i},$$

where *w_i_* is the width of region *i*, *A* and *B* are index sets for background and input regions with the relation B⊆A. If the widths of regions are all similar, *p*_2_ can be approximated as *p*_2_ ≈ *n*_bg_/*N*_bg_. Nevertheless, the binomial method has no restriction as the hypergeometric method where input regions must be perfect subsets of the background, therefore, the binomial method proposed in *rGREAT* is more general than the native hypergeometric method in *GREAT* for dealing with backgrounds.

In Supplementary File S1, we applied functional enrichment on a TFBS dataset by taking regions in different chromatin states as backgrounds. We found TFBSs are specifically enriched in more biological functions when taking enhancers as background than promoters.

### 2.4 Comparison of online and local GREAT

In Supplementary File S2, we compared GO gene sets used in the *GREAT* web service and in local *GREAT* analysis in *rGREAT*. We found GO gene sets in the *GREAT* web service are outdated and have inconsistencies compared to the newest ones. In comparison, local *GREAT* always uses the newest GO annotations from the *GO.db* package which is updated twice a year by the Bioconductor core team. Nevertheless, in Supplementary File S3, we compared the enrichment results from online *GREAT* and local *GREAT* with four TFBS datasets, and in general, the results from the two *GREAT* analyses are very consistent.

### 2.5 Different TSS annotations

The *GREAT* method depends on the locations of TSSs. Different sources may have different annotations of TSSs. great() supports four sources of TSSs, which are (1) the Bioconductor TxDb.*packages, e.g. the *TxDb.Hsapiens.UCSC.hg19.knownGene* package for humans, (2) RefSeq genes, e.g. the RefSeq *Select* or *Curated* subset, (3) GENCODE annotations (Frankish *et al.*, 2021) and (4) TSSs provided by *GREAT* itself. In Supplementary File S4, we demonstrated that the four TSS sources almost cover the same set of genes in the genome, but the exact locations of TSSs differ a lot. For example, the locations of GENCODE and RefSeq TSSs have a mean difference of 1255 bp and a median difference of 14 bp. Nevertheless, the inconsistency of TSS locations has very little effect on the enrichment results, mainly because the difference is ignorable compared to the scales of extended TSSs. In Supplementary File S4, we demonstrated with a given TFBS dataset that the enrichment results from the four TSS annotations are highly consistent.

### 2.6 GO gene sets from Ensembl BioMart

To facilitate analysis for non-model organisms, we have compiled gene annotations and GO gene sets for more than 600 organisms from the Ensembl BioMart web service (Kinsella *et al.*, 2011) with the package *biomaRt* (Durinck *et al.*, 2005). All the genes and gene sets are available in a separate Bioconductor package *BioMartGOGeneSets*. It is seamlessly integrated with *rGREAT* and will be updated regularly.

## 3 Conclusion

We developed the new R/Bioconductor package *rGREAT* for functional enrichment on genomic regions. *rGREAT* integrates a large number of gene set collections for many organisms. We believe it will be a useful tool for functional interpretations in genomics and epigenomics studies, especially for studies on non-model organisms.

## Supplementary Material

btac745_Supplementary_Data
