# Supplementary material for: rGREAT: an R/bioconductor package for functional enrichment on genomic regions
Source: Bioinformatics. 2022 Nov 17;39(1):btac745. doi: 10.1093/bioinformatics/btac745 (PMC9805586; doi:10.1093/bioinformatics/btac745)
Supplement: btac745_Supplementary_Data [file btac745_supplementary_data.zip › btac745_Supplementary_Data/suppl2_compare_GO.zip!/compare_GO.html]

Suppl 2. Compare GO annotations from online and local GREAT


# Suppl 2. Compare GO annotations from online and local GREAT

#### Zuguang Gu ( z.gu@dkfz.de )

#### 2022-10-04

According to the **GREAT** website, the GO gene sets they use were generated between 2011 and 2012. In this document, we are going to compare the GO gene sets integrated in GREAT web service and the GO gene sets integrated in local GREAT analysis.

**GREAT** does not provide files of gene sets they use, but in the result table from GREAT analysis, there is a column “Hyper\_Total\_Genes” which is the total number of genes in each gene set, which we can use to compare.

Total number of genes in gene sets is not affected by which input regions to use. Here we simply generate a random set of input regions.

```
library(rGREAT)
gr = randomRegions(genome = "hg19")
```

We first perform the online GREAT analysis and retrieve enrichment result for the GO:BP ontology.

```
job = submitGreatJob(gr)
tbl = getEnrichmentTables(job)
tb1 = tbl[["GO Biological Process"]]
```

Next we perform local GREAT analysis. We use the same TSS definition as online GREAT, but here GO gene sets are from the **GO.db** package (versio 3.14).

```
res2 = great(gr, "GO:BP", "GREAT:hg19", min_gene_set_size = 0)
tb2 = getEnrichmentTable(res2, min_region_hits = 0)
```

`tb1` and `tb2` contain the full set of GO terms under test, thus, we can compare the GO terms in the two sources:

```
library(eulerr)
plot(euler(list(GREAT = tb1$ID, "GO.db" = tb2$id)), quantities = T)
```

So basically, the two GO sources have very high agreement, but **GO.db** has more additional GO terms.

The absolute numbers of GO terms are less important because some very general GO terms might be filtered out by one source. For the ease of comparison, we take the common GO terms in the two sources:

```
rownames(tb1) = tb1$ID
rownames(tb2) = tb2$id

cn = intersect(rownames(tb1), rownames(tb2))
length(cn)
```

```
## [1] 12361
```

```
tb1 = tb1[cn, ]
tb2 = tb2[cn, ]
head(tb1)
```

```
##                    ID                                                  name
## GO:0002082 GO:0002082               regulation of oxidative phosphorylation
## GO:0048535 GO:0048535                                lymph node development
## GO:0031113 GO:0031113              regulation of microtubule polymerization
## GO:1902988 GO:1902988                       neurofibrillary tangle assembly
## GO:1905689 GO:1905689 positive regulation of diacylglycerol kinase activity
## GO:0006501 GO:0006501                         C-terminal protein lipidation
##            Binom_Genome_Fraction Binom_Expected Binom_Observed_Region_Hits
## GO:0002082          1.419547e-03     1.41954700                          7
## GO:0048535          1.555425e-03     1.55542500                          7
## GO:0031113          3.044336e-03     3.04433600                         10
## GO:1902988          5.184967e-05     0.05184967                          2
## GO:1905689          5.184967e-05     0.05184967                          2
## GO:0006501          9.898874e-03     9.89887400                         21
##            Binom_Fold_Enrichment Binom_Region_Set_Coverage Binom_Raw_PValue
## GO:0002082              4.931150                     0.007     0.0006654645
## GO:0048535              4.500378                     0.007     0.0011242450
## GO:0031113              3.284788                     0.010     0.0012010760
## GO:1902988             38.573050                     0.002     0.0012974110
## GO:1905689             38.573050                     0.002     0.0012974110
## GO:0006501              2.121453                     0.021     0.0013260290
##            Binom_Adjp_BH Hyper_Total_Genes Hyper_Expected
## GO:0002082             1                17     1.30141800
## GO:0048535             1                17     1.30141800
## GO:0031113             1                38     2.90905200
## GO:1902988             1                 1     0.07655399
## GO:1905689             1                 1     0.07655399
## GO:0006501             1                77     5.89465700
##            Hyper_Observed_Gene_Hits Hyper_Fold_Enrichment
## GO:0002082                        5              3.841964
## GO:0048535                        5              3.841964
## GO:0031113                        6              2.062528
## GO:1902988                        1             13.062680
## GO:1905689                        1             13.062680
## GO:0006501                       14              2.375032
##            Hyper_Gene_Set_Coverage Hyper_Term_Gene_Coverage Hyper_Raw_PValue
## GO:0002082            0.0035211270                0.2941176      0.007413595
## GO:0048535            0.0035211270                0.2941176      0.007413595
## GO:0031113            0.0042253520                0.1578947      0.066910920
## GO:1902988            0.0007042254                1.0000000      0.076553990
## GO:1905689            0.0007042254                1.0000000      0.076553990
## GO:0006501            0.0098591550                0.1818182      0.001941707
##            Hyper_Adjp_BH
## GO:0002082    0.20720282
## GO:0048535    0.20720282
## GO:0031113    0.67582418
## GO:1902988    0.67582418
## GO:1905689    0.67582418
## GO:0006501    0.08893289
```

```
head(tb2)
```

```
##                    id                                           description
## GO:0002082 GO:0002082               regulation of oxidative phosphorylation
## GO:0048535 GO:0048535                                lymph node development
## GO:0031113 GO:0031113              regulation of microtubule polymerization
## GO:1902988 GO:1902988                       neurofibrillary tangle assembly
## GO:1905689 GO:1905689 positive regulation of diacylglycerol kinase activity
## GO:0006501 GO:0006501                         C-terminal protein lipidation
##            genome_fraction observed_region_hits fold_enrichment     p_value
## GO:0002082    1.879025e-03                    6        3.505099 0.008189002
## GO:0048535    1.969298e-03                    7        3.901829 0.002491358
## GO:0031113    5.484272e-03                   13        2.601992 0.001940687
## GO:1902988    3.870682e-04                    2        5.671841 0.049287574
## GO:1905689    5.185067e-05                    2       42.340627 0.001079988
## GO:0006501    5.616932e-04                    1        1.954261 0.400612062
##            p_adjust mean_tss_dist observed_gene_hits gene_set_size
## GO:0002082        1        123496                  4            21
## GO:0048535        1        316468                  5            18
## GO:0031113        1        216944                  9            55
## GO:1902988        1         27406                  1             3
## GO:1905689        1         27406                  1             1
## GO:0006501        1        208760                  1             7
##            fold_enrichment_hyper p_value_hyper p_adjust_hyper
## GO:0002082              2.482979   0.072362294       0.716660
## GO:0048535              3.621011   0.009710746       0.257238
## GO:0031113              2.133104   0.023121265       0.436428
## GO:1902988              4.345213   0.212945955       1.000000
## GO:1905689             13.035638   0.076712778       0.716660
## GO:0006501              1.862234   0.428104957       1.000000
```

The column `"Hyper_Total_Genes"` in `tb1` and the column `"gene_set_size"` in `tb2` all correspond to the numbers of genes in GO gene sets. We can directly compare the two columns of values.

```
plot(tb1$Hyper_Total_Genes, tb2$gene_set_size,
    xlab = "online-GREAT", ylab = "GO.db", main = "Gene set sizes")
```

In general, the two vectors agrees very linearly.

Next we add a third source of GO gene sets, which is from MSigDB. Similarly, we perform local GREAT analysis and extract the enrichment table.

```
res3 = great(gr, "msigdb:C5:GO:BP", "GREAT:hg19", min_gene_set_size = 0)
tb3 = getEnrichmentTable(res3, min_region_hits = 0)
head(tb3)
```

```
##                                                           id genome_fraction
## 1                               GOBP_VASCULATURE_DEVELOPMENT    0.0953996716
## 2                            GOBP_BLOOD_VESSEL_MORPHOGENESIS    0.0833748197
## 3                                   GOBP_VIRAL_TRANSCRIPTION    0.0043821938
## 4 GOBP_POSITIVE_REGULATION_OF_ACTIVATED_T_CELL_PROLIFERATION    0.0017041350
## 5      GOBP_NEGATIVE_REGULATION_OF_OXIDATIVE_PHOSPHORYLATION    0.0008714775
## 6                           GOBP_HISTIDINE_METABOLIC_PROCESS    0.0009349203
##   observed_region_hits fold_enrichment      p_value p_adjust mean_tss_dist
## 1                  118        1.357740 0.0004724966        1        278428
## 2                  105        1.382407 0.0005416053        1        279017
## 3                   12        3.005878 0.0008714004        1        280938
## 4                    7        4.508953 0.0011107741        1        246115
## 5                    5        6.297895 0.0013547826        1        147390
## 6                    5        5.870526 0.0018368685        1        259345
##   observed_gene_hits gene_set_size fold_enrichment_hyper p_value_hyper
## 1                106           719              1.921805  3.835621e-11
## 2                 92           607              1.975747  1.821290e-10
## 3                  6            51              1.533604  1.942354e-01
## 4                  6            25              3.128553  1.004911e-02
## 5                  3             7              5.586702  1.246602e-02
## 6                  2            11              2.370116  2.044811e-01
##   p_adjust_hyper
## 1   4.895531e-08
## 2   1.992491e-07
## 3   8.422447e-01
## 4   1.968186e-01
## 5   2.194592e-01
## 6   8.515041e-01
```

In MSigDB, the IDs of GO gene sets are GO term names, thus we need to convert them to GO IDs:

```
library(GO.db)
lt = as.list(GOTERM)
map = sapply(lt, function(x) Term(x))
map = map[sapply(lt, function(x) Ontology(x) == "BP")]
map = toupper(map)
map = gsub(" ", "_", map)
map2 = structure(names(map), names = map)
new_rn = map2[ gsub("^GOBP_", "", tb3$id) ]
l = !is.na(new_rn)
tb3 = tb3[l, ]
rownames(tb3) = new_rn[l]
```

Next we take the common GO terms in the three sources:

```
cn = intersect(cn, rownames(tb3))
length(cn)
```

```
## [1] 5615
```

You may find the number of common GO terms becomes much smaller. It is because MSigDB does not contain the full set of GO terms. Some terms that are not informative are removed there.

```
tb1 = tb1[cn, ]
tb2 = tb2[cn, ]
tb3 = tb3[cn, ]
```

And we compare the gene set sizes in the three sources:

```
par(mfrow = c(1, 3))
max = max(tb1$Hyper_Total_Genes, tb2$gene_set_size, tb3$gene_set_size)
plot(tb1$Hyper_Total_Genes, tb2$gene_set_size, xlim = c(0, max), ylim = c(0, max),
    xlab = "online-GREAT", ylab = "GO.db", main = "Gene set sizes")
plot(tb1$Hyper_Total_Genes, tb3$gene_set_size, xlim = c(0, max), ylim = c(0, max),
    xlab = "online-GREAT", ylab = "MSigDB", main = "Gene set sizes")
plot(tb2$gene_set_size, tb3$gene_set_size, xlim = c(0, max), ylim = c(0, max),
    xlab = "GO.db", ylab = "MSigDB", main = "Gene set sizes")
```

So here we see GO gene sets from **GO.db** and MSigDb are almost identical (the third plot), while there are certain degrees of inconsistency between online GREAT and the other two (the first two plots).

Now we can make the conclusion that the GO gene sets in online GREAT are outdated and are not very consistent to the most up-to-date ones.

## Session info

```
sessionInfo()
```

```
## R version 4.2.0 (2022-04-22)
## Platform: x86_64-apple-darwin17.0 (64-bit)
## Running under: macOS Big Sur/Monterey 10.16
## 
## Matrix products: default
## BLAS:   /Library/Frameworks/R.framework/Versions/4.2/Resources/lib/libRblas.0.dylib
## LAPACK: /Library/Frameworks/R.framework/Versions/4.2/Resources/lib/libRlapack.dylib
## 
## locale:
## [1] C/UTF-8/C/C/C/C
## 
## attached base packages:
## [1] stats4    stats     graphics  grDevices utils     datasets  methods  
## [8] base     
## 
## other attached packages:
##  [1] GO.db_3.15.0         AnnotationDbi_1.58.0 Biobase_2.56.0      
##  [4] eulerr_6.1.1         rGREAT_1.99.7        GenomicRanges_1.48.0
##  [7] GenomeInfoDb_1.32.2  IRanges_2.30.0       S4Vectors_0.34.0    
## [10] BiocGenerics_0.42.0  knitr_1.39           colorout_1.2-2      
## 
## loaded via a namespace (and not attached):
##   [1] bitops_1.0-7                            
##   [2] matrixStats_0.62.0                      
##   [3] bit64_4.0.5                             
##   [4] doParallel_1.0.17                       
##   [5] filelock_1.0.2                          
##   [6] RColorBrewer_1.1-3                      
##   [7] progress_1.2.2                          
##   [8] httr_1.4.3                              
##   [9] tools_4.2.0                             
##  [10] bslib_0.4.0                             
##  [11] utf8_1.2.2                              
##  [12] R6_2.5.1                                
##  [13] DT_0.23                                 
##  [14] colorspace_2.0-3                        
##  [15] DBI_1.1.3                               
##  [16] GetoptLong_1.1.0                        
##  [17] tidyselect_1.1.2                        
##  [18] prettyunits_1.1.1                       
##  [19] bit_4.0.4                               
##  [20] curl_4.3.2                              
##  [21] compiler_4.2.0                          
##  [22] cli_3.3.0                               
##  [23] xml2_1.3.3                              
##  [24] DelayedArray_0.22.0                     
##  [25] rtracklayer_1.56.1                      
##  [26] sass_0.4.2                              
##  [27] rappdirs_0.3.3                          
##  [28] stringr_1.4.0                           
##  [29] digest_0.6.29                           
##  [30] Rsamtools_2.12.0                        
##  [31] rmarkdown_2.14                          
##  [32] XVector_0.36.0                          
##  [33] pkgconfig_2.0.3                         
##  [34] htmltools_0.5.3                         
##  [35] MatrixGenerics_1.8.1                    
##  [36] highr_0.9                               
##  [37] dbplyr_2.2.1                            
##  [38] fastmap_1.1.0                           
##  [39] htmlwidgets_1.5.4                       
##  [40] rlang_1.0.4                             
##  [41] GlobalOptions_0.1.2                     
##  [42] RSQLite_2.2.15                          
##  [43] shiny_1.7.2                             
##  [44] shape_1.4.6                             
##  [45] TxDb.Hsapiens.UCSC.hg19.knownGene_3.2.2 
##  [46] jquerylib_0.1.4                         
##  [47] BiocIO_1.6.0                            
##  [48] generics_0.1.3                          
##  [49] jsonlite_1.8.0                          
##  [50] BiocParallel_1.30.3                     
##  [51] dplyr_1.0.9                             
##  [52] RCurl_1.98-1.8                          
##  [53] magrittr_2.0.3                          
##  [54] GenomeInfoDbData_1.2.8                  
##  [55] Matrix_1.4-1                            
##  [56] Rcpp_1.0.9                              
##  [57] fansi_1.0.3                             
##  [58] lifecycle_1.0.1                         
##  [59] stringi_1.7.8                           
##  [60] yaml_2.3.5                              
##  [61] SummarizedExperiment_1.26.1             
##  [62] zlibbioc_1.42.0                         
##  [63] org.Hs.eg.db_3.15.0                     
##  [64] BiocFileCache_2.4.0                     
##  [65] grid_4.2.0                              
##  [66] blob_1.2.3                              
##  [67] promises_1.2.0.1                        
##  [68] parallel_4.2.0                          
##  [69] crayon_1.5.1                            
##  [70] lattice_0.20-45                         
##  [71] Biostrings_2.64.0                       
##  [72] GenomicFeatures_1.48.3                  
##  [73] circlize_0.4.16                         
##  [74] hms_1.1.1                               
##  [75] KEGGREST_1.36.3                         
##  [76] polylabelr_0.2.0                        
##  [77] pillar_1.8.0                            
##  [78] rjson_0.2.21                            
##  [79] codetools_0.2-18                        
##  [80] biomaRt_2.52.0                          
##  [81] XML_3.99-0.10                           
##  [82] glue_1.6.2                              
##  [83] evaluate_0.15                           
##  [84] httpuv_1.6.5                            
##  [85] foreach_1.5.2                           
##  [86] png_0.1-7                               
##  [87] vctrs_0.4.1                             
##  [88] polyclip_1.10-0                         
##  [89] purrr_0.3.4                             
##  [90] assertthat_0.2.1                        
##  [91] cachem_1.0.6                            
##  [92] xfun_0.31                               
##  [93] mime_0.12                               
##  [94] xtable_1.8-4                            
##  [95] restfulr_0.0.15                         
##  [96] later_1.3.0                             
##  [97] tibble_3.1.8                            
##  [98] TxDb.Hsapiens.UCSC.hg38.knownGene_3.15.0
##  [99] iterators_1.0.14                        
## [100] GenomicAlignments_1.32.1                
## [101] memoise_2.0.1                           
## [102] ellipsis_0.3.2
```
