# Supplementary material for: rGREAT: an R/bioconductor package for functional enrichment on genomic regions
Source: Bioinformatics. 2022 Nov 17;39(1):btac745. doi: 10.1093/bioinformatics/btac745 (PMC9805586; doi:10.1093/bioinformatics/btac745)
Supplement: btac745_Supplementary_Data [file btac745_supplementary_data.zip › btac745_Supplementary_Data/suppl3_compare_online_and_local.zip!/compare_online_and_local.html]

Suppl 3. Compare online and local GREAT


# Suppl 3. Compare online and local GREAT

#### Zuguang Gu ( z.gu@dkfz.de )

#### 2022-10-04

```
library(rGREAT)
library(eulerr)
```

In this document, we will compare the enrichment results from online GREAT and local GREAT. The four datasets are all from UCSC table browser. Parameters are:

```
clade = Mammal
genome = Human
assembly = GRCh37/hg19
group = Regulation
track = ENCODE 3 TFBS
table: A549 JUN, A549 ELF1, H1-hESC RXRA, GM12878 MYB
```

And in the “Retrieve and display data” section:

```
output format = BED - browser extensible data
```

Then click the button “get output”.

We first read the files into `GRanges` objects:

```
read_bed = function(f) {
    df = read.table(f)
    df = df[df[, 1] %in% paste0("chr", c(1:22, "X", "Y")), ]
    GRanges(seqnames = df[, 1], ranges = IRanges(df[, 2] + 1, df[, 3]))
}
grl = list()
grl$A549_JUN = read_bed("data/tb_encTfChipPkENCFF708LCH_A549_JUN_hg19.bed")
grl$A549_ELF1 = read_bed("data/tb_encTfChipPkENCFF533NIV_A549_ELF1_hg19.bed")
grl$H1_hESC_RXRA = read_bed("data/tb_encTfChipPkENCFF369JAI_H1_hESC_RXRA_hg19.bed")
grl$GM12878_MYB = read_bed("data/tb_encTfChipPkENCFF215YWS_GM12878_MYB_hg19.bed")
sapply(grl, length)
```

```
##     A549_JUN    A549_ELF1 H1_hESC_RXRA  GM12878_MYB 
##         1726        11577         2092         3748
```

## A549\_JUN (1726 input regions)

Apply both online and local GREAT analysis. Note online GREAT exclude gap regions, and in local GREAT, by default gap regions are removed as well.

```
gr = grl$A549_JUN
job = submitGreatJob(gr)
tbl = getEnrichmentTables(job)
tb1 = tbl[["GO Biological Process"]]

res = great(gr, "GO:BP", "hg19")
tb2 = getEnrichmentTable(res)
```

`tb1` and `tb2` contain the full table of all GO terms under test. First we take the common GO terms in the two result tables.

```
cn = intersect(tb1$ID, tb2$id)
length(cn)
```

```
## [1] 4567
```

```
rownames(tb1) = tb1$ID
rownames(tb2) = tb2$id
tb1 = tb1[cn, ]
tb2 = tb2[cn, ]
```

The significant GO terms from the two tables.

```
lt2 = list(online = tb1$ID[tb1$Binom_Adjp_BH < 0.001],
          local = tb2$id[tb2$p_adjust < 0.001])
plot(euler(lt2), quantities = TRUE, main = "A549_JUN")
```

Next we compare the observed region hits and fold enrichment in the two results.

```
par(mfrow = c(1, 2))
plot(tb1$Binom_Observed_Region_Hits, tb2$observed_region_hits, pch = 16, col = "#00000010",
    xlab = "online GREAT", ylab = "local GREAT", main = "Observed region hits")
plot(tb1$Binom_Fold_Enrichment, tb2$fold_enrichment, pch = 16, col = "#00000010",
    xlab = "online GREAT", ylab = "local GREAT", main = "Fold enrichment")
```

Next we compare the two significant GO term lists by clustering them into groups.

```
lt3 = list(online = data.frame(id = tb1$ID, p_adjust = tb1$Binom_Adjp_BH), 
           local = data.frame(id = tb2$id, p_adjust = tb2$p_adjust))
library(simplifyEnrichment)
se_opt$verbose = FALSE
simplifyGOFromMultipleLists(lt3, padj_cutoff = 0.001)
```

## A549\_ELF1 (11577 input regions)

Apply both online and local GREAT analysis:

```
gr = grl$A549_ELF1
job = submitGreatJob(gr)
tbl = getEnrichmentTables(job)
tb1 = tbl[["GO Biological Process"]]

res = great(gr, "GO:BP", "hg19")
tb2 = getEnrichmentTable(res)
```

`tb1` and `tb2` contain the full table of all GO terms under test. First we take the common GO terms in the two result tables.

```
cn = intersect(tb1$ID, tb2$id)
length(cn)
```

```
## [1] 7536
```

```
rownames(tb1) = tb1$ID
rownames(tb2) = tb2$id
tb1 = tb1[cn, ]
tb2 = tb2[cn, ]
```

The significant GO terms from the two tables.

```
lt2 = list(online = tb1$ID[tb1$Binom_Adjp_BH < 0.001],
          local = tb2$id[tb2$p_adjust < 0.001])
plot(euler(lt2), quantities = TRUE, main = "A549_ELF1")
```

Next we compare the observed region hits and fold enrichment in the two results.

```
par(mfrow = c(1, 2))
plot(tb1$Binom_Observed_Region_Hits, tb2$observed_region_hits, pch = 16, col = "#00000010",
    xlab = "online GREAT", ylab = "local GREAT", main = "Observed region hits")
plot(tb1$Binom_Fold_Enrichment, tb2$fold_enrichment, pch = 16, col = "#00000010",
    xlab = "online GREAT", ylab = "local GREAT", main = "Fold enrichment")
```

Next we compare the two significant GO term lists by clustering them into groups.

```
lt3 = list(online = data.frame(id = tb1$ID, p_adjust = tb1$Binom_Adjp_BH), 
           local = data.frame(id = tb2$id, p_adjust = tb2$p_adjust))
library(simplifyEnrichment)
se_opt$verbose = FALSE
simplifyGOFromMultipleLists(lt3, padj_cutoff = 0.001)
```

## H1\_hESC\_RXRA (2092 input regions)

Apply both online and local GREAT analysis:

```
gr = grl$H1_hESC_RXRA
job = submitGreatJob(gr)
tbl = getEnrichmentTables(job)
tb1 = tbl[["GO Biological Process"]]

res = great(gr, "GO:BP", "hg19")
tb2 = getEnrichmentTable(res)
```

`tb1` and `tb2` contain the full table of all GO terms under test. First we take the common GO terms in the two result tables.

```
cn = intersect(tb1$ID, tb2$id)
length(cn)
```

```
## [1] 4689
```

```
rownames(tb1) = tb1$ID
rownames(tb2) = tb2$id
tb1 = tb1[cn, ]
tb2 = tb2[cn, ]
```

The significant GO terms from the two tables.

```
lt2 = list(online = tb1$ID[tb1$Binom_Adjp_BH < 0.001],
          local = tb2$id[tb2$p_adjust < 0.001])
plot(euler(lt2), quantities = TRUE, main = "H1_hESC_RXRA")
```

Next we compare the observed region hits and fold enrichment in the two results.

```
par(mfrow = c(1, 2))
plot(tb1$Binom_Observed_Region_Hits, tb2$observed_region_hits, pch = 16, col = "#00000010",
    xlab = "online GREAT", ylab = "local GREAT", main = "Observed region hits")
plot(tb1$Binom_Fold_Enrichment, tb2$fold_enrichment, pch = 16, col = "#00000010",
    xlab = "online GREAT", ylab = "local GREAT", main = "Fold enrichment")
```

Next we compare the two significant GO term lists by clustering them into groups.

```
lt3 = list(online = data.frame(id = tb1$ID, p_adjust = tb1$Binom_Adjp_BH), 
           local = data.frame(id = tb2$id, p_adjust = tb2$p_adjust))
library(simplifyEnrichment)
se_opt$verbose = FALSE
simplifyGOFromMultipleLists(lt3, padj_cutoff = 0.001)
```

## GM12878\_MYB (3748 input regions)

Apply both online and local GREAT analysis:

```
gr = grl$GM12878_MYB
job = submitGreatJob(gr)
tbl = getEnrichmentTables(job)
tb1 = tbl[["GO Biological Process"]]

res = great(gr, "GO:BP", "hg19")
tb2 = getEnrichmentTable(res)
```

`tb1` and `tb2` contain the full table of all GO terms under test. First we take the common GO terms in the two result tables.

```
cn = intersect(tb1$ID, tb2$id)
length(cn)
```

```
## [1] 5740
```

```
rownames(tb1) = tb1$ID
rownames(tb2) = tb2$id
tb1 = tb1[cn, ]
tb2 = tb2[cn, ]
```

The significant GO terms from the two tables.

```
lt2 = list(online = tb1$ID[tb1$Binom_Adjp_BH < 0.001],
          local = tb2$id[tb2$p_adjust < 0.001])
plot(euler(lt2), quantities = TRUE, main = "GM12878_MYB")
```

Next we compare the observed region hits and fold enrichment in the two results.

```
par(mfrow = c(1, 2))
plot(tb1$Binom_Observed_Region_Hits, tb2$observed_region_hits, pch = 16, col = "#00000010",
    xlab = "online GREAT", ylab = "local GREAT", main = "Observed region hits")
plot(tb1$Binom_Fold_Enrichment, tb2$fold_enrichment, pch = 16, col = "#00000010",
    xlab = "online GREAT", ylab = "local GREAT", main = "Fold enrichment")
```

Next we compare the two significant GO term lists by clustering them into groups.

```
lt3 = list(online = data.frame(id = tb1$ID, p_adjust = tb1$Binom_Adjp_BH), 
           local = data.frame(id = tb2$id, p_adjust = tb2$p_adjust))
library(simplifyEnrichment)
se_opt$verbose = FALSE
simplifyGOFromMultipleLists(lt3, padj_cutoff = 0.001)
```

## Session info

```
sessionInfo()
```

```
## R version 4.2.0 (2022-04-22)
## Platform: x86_64-apple-darwin17.0 (64-bit)
## Running under: macOS Big Sur/Monterey 10.16
## 
## Matrix products: default
## BLAS:   /Library/Frameworks/R.framework/Versions/4.2/Resources/lib/libRblas.0.dylib
## LAPACK: /Library/Frameworks/R.framework/Versions/4.2/Resources/lib/libRlapack.dylib
## 
## locale:
## [1] C/UTF-8/C/C/C/C
## 
## attached base packages:
## [1] grid      stats4    stats     graphics  grDevices utils     datasets 
## [8] methods   base     
## 
## other attached packages:
##  [1] simplifyEnrichment_1.7.2 eulerr_6.1.1             rGREAT_1.99.7           
##  [4] GenomicRanges_1.48.0     GenomeInfoDb_1.32.2      IRanges_2.30.0          
##  [7] S4Vectors_0.34.0         BiocGenerics_0.42.0      knitr_1.39              
## [10] colorout_1.2-2          
## 
## loaded via a namespace (and not attached):
##   [1] colorspace_2.0-3                        
##   [2] rjson_0.2.21                            
##   [3] ellipsis_0.3.2                          
##   [4] circlize_0.4.16                         
##   [5] markdown_1.1                            
##   [6] XVector_0.36.0                          
##   [7] GlobalOptions_0.1.2                     
##   [8] gridtext_0.1.4                          
##   [9] clue_0.3-61                             
##  [10] DT_0.23                                 
##  [11] bit64_4.0.5                             
##  [12] AnnotationDbi_1.58.0                    
##  [13] fansi_1.0.3                             
##  [14] xml2_1.3.3                              
##  [15] codetools_0.2-18                        
##  [16] doParallel_1.0.17                       
##  [17] cachem_1.0.6                            
##  [18] GOSemSim_2.22.0                         
##  [19] polyclip_1.10-0                         
##  [20] jsonlite_1.8.0                          
##  [21] Cairo_1.6-0                             
##  [22] Rsamtools_2.12.0                        
##  [23] cluster_2.1.3                           
##  [24] GO.db_3.15.0                            
##  [25] dbplyr_2.2.1                            
##  [26] png_0.1-7                               
##  [27] shiny_1.7.2                             
##  [28] compiler_4.2.0                          
##  [29] httr_1.4.3                              
##  [30] assertthat_0.2.1                        
##  [31] Matrix_1.4-1                            
##  [32] fastmap_1.1.0                           
##  [33] TxDb.Hsapiens.UCSC.hg19.knownGene_3.2.2 
##  [34] cli_3.3.0                               
##  [35] later_1.3.0                             
##  [36] htmltools_0.5.3                         
##  [37] prettyunits_1.1.1                       
##  [38] tools_4.2.0                             
##  [39] NLP_0.2-1                               
##  [40] glue_1.6.2                              
##  [41] GenomeInfoDbData_1.2.8                  
##  [42] dplyr_1.0.9                             
##  [43] rappdirs_0.3.3                          
##  [44] Rcpp_1.0.9                              
##  [45] slam_0.1-50                             
##  [46] TxDb.Hsapiens.UCSC.hg38.knownGene_3.15.0
##  [47] Biobase_2.56.0                          
##  [48] jquerylib_0.1.4                         
##  [49] vctrs_0.4.1                             
##  [50] Biostrings_2.64.0                       
##  [51] rtracklayer_1.56.1                      
##  [52] iterators_1.0.14                        
##  [53] xfun_0.31                               
##  [54] polylabelr_0.2.0                        
##  [55] stringr_1.4.0                           
##  [56] mime_0.12                               
##  [57] lifecycle_1.0.1                         
##  [58] restfulr_0.0.15                         
##  [59] XML_3.99-0.10                           
##  [60] org.Hs.eg.db_3.15.0                     
##  [61] zlibbioc_1.42.0                         
##  [62] hms_1.1.1                               
##  [63] promises_1.2.0.1                        
##  [64] MatrixGenerics_1.8.1                    
##  [65] parallel_4.2.0                          
##  [66] SummarizedExperiment_1.26.1             
##  [67] RColorBrewer_1.1-3                      
##  [68] ComplexHeatmap_2.13.2                   
##  [69] yaml_2.3.5                              
##  [70] curl_4.3.2                              
##  [71] memoise_2.0.1                           
##  [72] sass_0.4.2                              
##  [73] biomaRt_2.52.0                          
##  [74] stringi_1.7.8                           
##  [75] RSQLite_2.2.15                          
##  [76] highr_0.9                               
##  [77] BiocIO_1.6.0                            
##  [78] foreach_1.5.2                           
##  [79] GenomicFeatures_1.48.3                  
##  [80] filelock_1.0.2                          
##  [81] BiocParallel_1.30.3                     
##  [82] shape_1.4.6                             
##  [83] rlang_1.0.4                             
##  [84] pkgconfig_2.0.3                         
##  [85] matrixStats_0.62.0                      
##  [86] bitops_1.0-7                            
##  [87] evaluate_0.15                           
##  [88] lattice_0.20-45                         
##  [89] purrr_0.3.4                             
##  [90] GenomicAlignments_1.32.1                
##  [91] htmlwidgets_1.5.4                       
##  [92] bit_4.0.4                               
##  [93] tidyselect_1.1.2                        
##  [94] magrittr_2.0.3                          
##  [95] R6_2.5.1                                
##  [96] magick_2.7.3                            
##  [97] generics_0.1.3                          
##  [98] DelayedArray_0.22.0                     
##  [99] DBI_1.1.3                               
## [100] pillar_1.8.0                            
## [101] proxyC_0.2.4                            
## [102] KEGGREST_1.36.3                         
## [103] RCurl_1.98-1.8                          
## [104] tibble_3.1.8                            
## [105] crayon_1.5.1                            
## [106] utf8_1.2.2                              
## [107] BiocFileCache_2.4.0                     
## [108] rmarkdown_2.14                          
## [109] GetoptLong_1.1.0                        
## [110] progress_1.2.2                          
## [111] blob_1.2.3                              
## [112] digest_0.6.29                           
## [113] tm_0.7-8                                
## [114] xtable_1.8-4                            
## [115] httpuv_1.6.5                            
## [116] RcppParallel_5.1.5                      
## [117] bslib_0.4.0
```
