# Supplementary material for: rGREAT: an R/bioconductor package for functional enrichment on genomic regions
Source: Bioinformatics. 2022 Nov 17;39(1):btac745. doi: 10.1093/bioinformatics/btac745 (PMC9805586; doi:10.1093/bioinformatics/btac745)
Supplement: btac745_Supplementary_Data [file btac745_supplementary_data.zip › btac745_Supplementary_Data/suppl4_compare_tss.zip!/compare_tss.html]

Suppl 4. Compare different TSS sources


# Suppl 4. Compare different TSS sources

#### Zuguang Gu ( z.gu@dkfz.de )

#### 2022-10-04

`great()` supports various sources to obtain gene TSS. The sources can be one of:

- a `TxDb.*` package such as **TxDb.Hsapiens.UCSC.hg38.knownGene**,
- RefSeq database (mainly the “RefSeq Select” subset (see https://genome.ucsc.edu/cgi-bin/hgTrackUi?db=hg38&g=refSeqComposite for different subsets of RefSeq),
- Gencode annotation,
- TSS provided by GREAT itself.

In this document, we will compare different TSS sources and their influence on GREAT enrichment analysis.

We use human genome hg38 here because there will be gene ID conversions (e.g. from Ensembl ID to Entrez ID for Gencode annotation), using the newest genome annotation version will reduce the inconsistency between different sources.

The helper function `getTSS()` extracts TSS from a specific source. Note genes in the four sources are protein-coding genes.

```
library(rGREAT)

tss_txdb = getTSS("TxDb.Hsapiens.UCSC.hg38.knownGene")
tss_gencode = getTSS("gencode_v40")
tss_refseq = getTSS("refseq:hg38")
tss_great = getTSS("great:hg38")
```

Gene IDs in `tss_gencode` are Ensembl gene IDs, and gene IDs in `tss_great` are gene symbols. We convert them to Entrez gene IDs.

```
library(org.Hs.eg.db)

map = unlist(as.list(org.Hs.egENSEMBL2EG))
new_gene_id = map[tss_gencode$gene_id]
tss_gencode$gene_id[!is.na(new_gene_id)] = new_gene_id[!is.na(new_gene_id)]

map = unlist(as.list(org.Hs.egSYMBOL2EG))
new_gene_id = map[tss_great$gene_id]
tss_great$gene_id[!is.na(new_gene_id)] = new_gene_id[!is.na(new_gene_id)]
```

We put all TSS objects into a single list:

```
tss_lt = list(
    txdb_known_gene = tss_txdb,
    gencode = tss_gencode,
    refseq = tss_refseq,
    great = tss_great
)
tss_lt = lapply(tss_lt, sort)
```

We first look at the overlap of genes. It basically shows all five sources almost contain the same set of genes.

```
library(ComplexHeatmap)
lt = lapply(tss_lt, function(x) {
    unique(x$gene_id)
})
cm = make_comb_mat(lt)
UpSet(cm, column_title = "Number of genes")
```

Next we look at the overlap of TSS with their exact positions. There are quite a large disagreement between different TSS sources.

```
lt = lapply(tss_lt, function(x) {
    unique(paste0(strand(x), seqnames(x), ":", start(x)))
})
cm = make_comb_mat(lt)
UpSet(cm, column_title = "Number of TSS (with their exact positions)")
```

Next we compare difference of TSS locations in different sources. We first take the common genes in all four sources.

```
tss_lt2 = lapply(tss_lt, function(x) {
    tb = table(x$gene_id)
    dp = names(tb[which(tb == 1)])
    x = x[x$gene_id %in% dp]
    names(x) = x$gene_id
    x
})
cn = tss_lt2[[1]]$gene_id
for(i in 2:length(tss_lt2)) {
    cn = intersect(cn, tss_lt2[[i]]$gene_id)
}
length(cn)
```

```
## [1] 17113
```

```
tss_lt2 = lapply(tss_lt2, function(x) x[cn])
```

Next we perform pairwise comparisons for every pair of TSS sources.

```
library(GetoptLong)
compare_tss_pos = function(tss1, tss2, name1, name2, ...) {
    d1 = start(tss1)
    d2 = start(tss2)

    diff = abs(d1 - d2)
    
    v = numeric()
    v["0"] = sum(diff == 0)
    v["1-5"] = sum(diff >= 1 & diff <= 5)
    v["6-10"] = sum(diff >= 6 & diff <= 10)
    v["11-50"] = sum(diff >= 11 & diff <= 50)
    v["51-500"] = sum(diff >= 51 & diff <= 500)
    v["501-5kb"] = sum(diff >= 501 & diff <= 5000)
    v["5kb-50kb"] = sum(diff >= 5001 & diff <= 50000)
    v[">50kb"] = sum(diff >= 50001)

    barplot(v, ylab = "Number of TSS", 
        main = qq("TSS dist_diff, @{name1} and @{name2}\nmean (trim 0.05) = @{round(mean(diff, trim = 0.05))}bp, median = @{median(diff)}bp"), 
        las = 3, ...)
}

par(mfrow = c(4, 4))
for(i in 1:4) {
    for(j in 1:4) {
        if(i == j) {
            plot(c(0, 1), c(0, 1), type = "n", axes = FALSE, ann = FALSE)
            text(0.5, 0.5, names(tss_lt2)[i], cex = 1.5)
        } else {
            compare_tss_pos(tss_lt2[[j]], tss_lt2[[i]], names(tss_lt2)[j], names(tss_lt2)[i], ylim = c(0, 16000))
        }
    }
}
```

Top 10 TSS which the highest variability of their positions:

```
library(matrixStats)
pos_mat = do.call(cbind, lapply(tss_lt2, start))
v = rowSds(pos_mat)
ind = order(v, decreasing = TRUE)[1:10]
pos_mat2 = data.frame("chr" = as.vector(seqnames(tss_lt2[[1]])), pos_mat, Entrez_ID = tss_lt2[[1]]$gene_id)
pos_mat2 = pos_mat2[ind, ]

library(org.Hs.eg.db)
map = unlist(as.list(org.Hs.egSYMBOL))

pos_mat2$Entrez_ID = qq("[@{pos_mat2$Entrez_ID}](https://www.genecards.org/cgi-bin/carddisp.pl?gene=@{map[pos_mat2$Entrez_ID]}#genomic_location)", collapse = FALSE)
kable(pos_mat2, row.names = FALSE)
```

| chr | txdb\_known\_gene | gencode | refseq | great | Entrez\_ID |
| --- | --- | --- | --- | --- | --- |
| chr1 | 58546734 | 58546734 | 57424060 | 57424058 | 1600 |
| chrX | 37349330 | 38561542 | 38561542 | 38561370 | 7102 |
| chr11 | 41459773 | 41459773 | 41459652 | 40294115 | 57689 |
| chr3 | 75906695 | 75906695 | 77040099 | 75906695 | 6092 |
| chr8 | 31639222 | 31639222 | 32548311 | 32548635 | 3084 |
| chr17 | 34157294 | 34174964 | 33293295 | 33292989 | 40 |
| chr10 | 55627942 | 55627942 | 54801231 | 54801292 | 65217 |
| chr16 | 73891871 | 73891871 | 73048128 | 73131095 | 463 |
| chr16 | 5239802 | 5239802 | 6019024 | 6019703 | 54715 |
| chr3 | 24687887 | 24687887 | 25428263 | 25428311 | 5915 |

## Influence on GREAT enrichment

Although TSSs have different positions in different sources, they are located quite closely. We next check whether the inconsistency of TSS positions affects the GREAT enrichment analysis.

In the next example, we use a dataset from UCSC table browser. The parameters are as follows:

```
clade = Mammal
genome = Human
assembly = GRCh38/hg38
group = Regulation
track = TF ChIP
table = A549 MYC (encTfChipPkENCFF542GMN)
```

Similarly, we perform local GREAT with four different TSS sources.

```
df = read.table("data/A549_MYC_encTfChipPkENCFF542GMN_hg38.bed")
df = df[df[, 1] %in% paste0("chr", c(1:22, "X", "Y")), ]
gr = GRanges(seqnames = df[, 1], ranges = IRanges(df[, 2]+1, df[, 3]))
res_txdb = great(gr, "GO:BP", "TxDb.Hsapiens.UCSC.hg38.knownGene", min_gene_set_size = 0)
res_gencode = great(gr, "GO:BP", "gencode_v40", min_gene_set_size = 0)
res_refseq = great(gr, "GO:BP", "refseq:hg38", min_gene_set_size = 0)
res_great = great(gr, "GO:BP", "great:hg38", min_gene_set_size = 0)

res_list = list(
    txdb_known_gene = res_txdb,
    gencode = res_gencode,
    refseq = res_refseq,
    great = res_great
)
```

We check the overlap of significant GO terms:

```
tb_list = lapply(res_list, function(x) getEnrichmentTable(x))

lt = lapply(tb_list, function(x) {
    x$id[x$p_adjust < 0.01]
})
cm = make_comb_mat(lt)
UpSet(cm, column_title = "Number of significant GO terms (FDR < 0.01)")
```

```
tb_list = lapply(res_list, function(x) getEnrichmentTable(x))
cn = intersect(tb_list[[1]]$id, intersect(tb_list[[2]]$id, intersect(tb_list[[3]]$id, tb_list[[4]]$id)))

vl = lapply(tb_list, function(x) {
    rownames(x) = x$id
    log2(x[cn, "fold_enrichment"])
})
par(mfrow = c(4, 4))
for(i in 1:4) {
    for(j in 1:4) {
        if(i == j) {
            plot(c(0, 1), c(0, 1), type = "n", axes = FALSE, ann = FALSE)
            text(0.5, 0.5, names(vl)[i], cex = 1.5)
        } else {
            plot(vl[[j]], vl[[i]], xlab = names(vl)[j], ylab = names(vl)[i], pch = 16, 
                col = "#00000020", main = "log2(Fold enrichment)",
                xlim = c(-6, 6), ylim = c(-6, 6))
        }
    }
}
```

The results shows the enrichments are very consistent for the four different TSS sources.

## Session info

```
sessionInfo()
```

```
## R version 4.2.0 (2022-04-22)
## Platform: x86_64-apple-darwin17.0 (64-bit)
## Running under: macOS Big Sur/Monterey 10.16
## 
## Matrix products: default
## BLAS:   /Library/Frameworks/R.framework/Versions/4.2/Resources/lib/libRblas.0.dylib
## LAPACK: /Library/Frameworks/R.framework/Versions/4.2/Resources/lib/libRlapack.dylib
## 
## locale:
## [1] C/UTF-8/C/C/C/C
## 
## attached base packages:
## [1] grid      stats4    stats     graphics  grDevices utils     datasets 
## [8] methods   base     
## 
## other attached packages:
##  [1] matrixStats_0.62.0    GetoptLong_1.1.0      ComplexHeatmap_2.13.2
##  [4] org.Hs.eg.db_3.15.0   AnnotationDbi_1.58.0  Biobase_2.56.0       
##  [7] rGREAT_1.99.7         GenomicRanges_1.48.0  GenomeInfoDb_1.32.2  
## [10] IRanges_2.30.0        S4Vectors_0.34.0      BiocGenerics_0.42.0  
## [13] knitr_1.39            colorout_1.2-2       
## 
## loaded via a namespace (and not attached):
##   [1] bitops_1.0-7                            
##   [2] bit64_4.0.5                             
##   [3] doParallel_1.0.17                       
##   [4] filelock_1.0.2                          
##   [5] RColorBrewer_1.1-3                      
##   [6] progress_1.2.2                          
##   [7] httr_1.4.3                              
##   [8] tools_4.2.0                             
##   [9] bslib_0.4.0                             
##  [10] utf8_1.2.2                              
##  [11] R6_2.5.1                                
##  [12] DT_0.23                                 
##  [13] colorspace_2.0-3                        
##  [14] DBI_1.1.3                               
##  [15] tidyselect_1.1.2                        
##  [16] prettyunits_1.1.1                       
##  [17] bit_4.0.4                               
##  [18] curl_4.3.2                              
##  [19] compiler_4.2.0                          
##  [20] cli_3.3.0                               
##  [21] Cairo_1.6-0                             
##  [22] xml2_1.3.3                              
##  [23] DelayedArray_0.22.0                     
##  [24] rtracklayer_1.56.1                      
##  [25] sass_0.4.2                              
##  [26] rappdirs_0.3.3                          
##  [27] stringr_1.4.0                           
##  [28] digest_0.6.29                           
##  [29] Rsamtools_2.12.0                        
##  [30] rmarkdown_2.14                          
##  [31] XVector_0.36.0                          
##  [32] pkgconfig_2.0.3                         
##  [33] htmltools_0.5.3                         
##  [34] MatrixGenerics_1.8.1                    
##  [35] highr_0.9                               
##  [36] dbplyr_2.2.1                            
##  [37] fastmap_1.1.0                           
##  [38] htmlwidgets_1.5.4                       
##  [39] rlang_1.0.4                             
##  [40] GlobalOptions_0.1.2                     
##  [41] RSQLite_2.2.15                          
##  [42] shiny_1.7.2                             
##  [43] shape_1.4.6                             
##  [44] TxDb.Hsapiens.UCSC.hg19.knownGene_3.2.2 
##  [45] jquerylib_0.1.4                         
##  [46] BiocIO_1.6.0                            
##  [47] generics_0.1.3                          
##  [48] jsonlite_1.8.0                          
##  [49] BiocParallel_1.30.3                     
##  [50] dplyr_1.0.9                             
##  [51] RCurl_1.98-1.8                          
##  [52] magrittr_2.0.3                          
##  [53] GO.db_3.15.0                            
##  [54] GenomeInfoDbData_1.2.8                  
##  [55] Matrix_1.4-1                            
##  [56] Rcpp_1.0.9                              
##  [57] fansi_1.0.3                             
##  [58] lifecycle_1.0.1                         
##  [59] stringi_1.7.8                           
##  [60] yaml_2.3.5                              
##  [61] SummarizedExperiment_1.26.1             
##  [62] zlibbioc_1.42.0                         
##  [63] BiocFileCache_2.4.0                     
##  [64] blob_1.2.3                              
##  [65] promises_1.2.0.1                        
##  [66] parallel_4.2.0                          
##  [67] crayon_1.5.1                            
##  [68] lattice_0.20-45                         
##  [69] Biostrings_2.64.0                       
##  [70] GenomicFeatures_1.48.3                  
##  [71] circlize_0.4.16                         
##  [72] hms_1.1.1                               
##  [73] KEGGREST_1.36.3                         
##  [74] magick_2.7.3                            
##  [75] pillar_1.8.0                            
##  [76] rjson_0.2.21                            
##  [77] codetools_0.2-18                        
##  [78] biomaRt_2.52.0                          
##  [79] XML_3.99-0.10                           
##  [80] glue_1.6.2                              
##  [81] evaluate_0.15                           
##  [82] httpuv_1.6.5                            
##  [83] foreach_1.5.2                           
##  [84] png_0.1-7                               
##  [85] vctrs_0.4.1                             
##  [86] purrr_0.3.4                             
##  [87] clue_0.3-61                             
##  [88] assertthat_0.2.1                        
##  [89] cachem_1.0.6                            
##  [90] xfun_0.31                               
##  [91] mime_0.12                               
##  [92] xtable_1.8-4                            
##  [93] restfulr_0.0.15                         
##  [94] later_1.3.0                             
##  [95] tibble_3.1.8                            
##  [96] TxDb.Hsapiens.UCSC.hg38.knownGene_3.15.0
##  [97] iterators_1.0.14                        
##  [98] GenomicAlignments_1.32.1                
##  [99] memoise_2.0.1                           
## [100] cluster_2.1.3                           
## [101] ellipsis_0.3.2
```
